# Vestibular Rehabilitation Thinking Beyond Benign Paroxysmal Positional Vertigo: Inference in a Rare Case of Oculocutaneous Albinism

**DOI:** 10.7759/cureus.30452

**Published:** 2022-10-19

**Authors:** Divya M Badjate, Rakesh K Kovela, Pallavi Harjpal, Shwetambari V Morghade

**Affiliations:** 1 Department of Physiotherapy, Ravi Nair Physiotherapy College, Datta Meghe Institute of Medical Sciences, Wardha, IND; 2 Department of Physiotherapy, Nitte Institute of Physiotherapy, Nitte University, Mangalore, IND; 3 Department of Neuro Physiotherapy, Ravi Nair Physiotherapy College, Datta Meghe Institute of Medical Sciences, Wardha, IND

**Keywords:** case report, vestibular rehabilitation, physical therapy rehabilitation, dizziness handicap inventory for patient caregivers (dhi-pc), dizziness, oculocutaneous albinism, nystagmus

## Abstract

Albinism is a group of heritable illnesses defined by a lack or loss of melanin in tissues originating from the ectoderm (most notably the skin, hair, and eyes). The most common kind of albinism is oculocutaneous albinism (OCA). Clinical evidence of less pigmentation of the hair and skin, as well as the characteristic ocular symptoms, are used to diagnose OCA. Nystagmus is one of the impacts of albinism on the eyes. Nystagmus is a term for involuntary ocular movements that are usually conjugate and rhythmic. Almost always, vertigo, dizziness, and loss of balance occur when nystagmus is accompanied by a condition of the inner ear's vestibular system or the brain. Nystagmus, which is induced by the rotation of an optokinetic drum or the rotation of the body in space, aids in visual maintenance. This case report describes the case of a 10-year-old male child with nystagmus associated with albinism, with typical complaints of dizziness that are scored on a Vanderbilt Pediatric Dizziness Handicap Inventory for Patient Caregivers (DHI-PC). Vestibular rehabilitation for nystagmus aids gaze stabilization, ocular muscle strength improvement, and vestibular function maintenance. The patient recovered with well-planned vestibular rehabilitation, which included gaze stability exercises, Cawthorne-Cooksey exercises, habituation exercises, eyeball resistance exercises, eye-hand coordination exercises, and parent education and home exercise programs.

## Introduction

Albinism is a class of heritable conditions marked by a deficiency or loss of melanin in ectoderm-derived structures (primarily the skin, hair, and eyes), resulting in noticeable hypopigmentation [1]. The most frequent kind of albinism is oculocutaneous albinism (OCA). OCA is a morphologically related group of genetic disorders characterized by abnormal melanin production. The most noticeable results, as the name suggests, are in the eyes and skin [2]. OCA refers to four autosomal recessive abnormalities typified by less pigmentation of the hair and skin, as well as the eyes, as a result of a complete loss or reduction in melanin production in the melanocytes [3]. Nystagmus is one of the impacts of albinism on the eyes [4]. Albinism is a hereditary disorder that impacts one in every 17,000 people. This suggests that one in every 70 people has the OCA gene [5]. Clinical evidence of less pigmentation of the hair and skin, as well as characteristic ocular symptoms, are used to diagnose OCA [6]. Fixation preference testing, such as Teller acuity cards, has been essential to analyzing vision. Visual evoked potential (VEP) testing is a ground-breaking method for determining visual acuity in people with pre-verbal albinism [7].

Nystagmus is a multifaceted visual impairment that has been connected to albinism [8]. Involuntary, frequently conjugate, and often rhythmic ocular movements are referred to as nystagmus [9]. The vestibule-ocular system stabilizes vision during head movement. One of the first picture stabilization control systems to be created was the vestibulo-ocular reflex (VOR) [10]. Some forms of nystagmus are completely natural. As a result, nystagmus due to the rotation of an optokinetic drum or the rotation of the body in space aids in maintaining eyesight. In pathological nystagmus, the rapid phase of nystagmus is commonly utilized to determine the direction of nystagmus [11]. A translucent iris, a transparent macula, and nystagmus all result in a decrease in quality of life [4]. Almost always, vertigo, dizziness, and loss of balance occur when nystagmus is accompanied by a condition of the inner ear's vestibular system or the brain. Nystagmus is commonly associated with hazy vision as well as jumping vision [1]. The most prevalent oculomotor abnormality found in individuals with dizziness in primary gaze is spontaneous nystagmus [12]. 4-Aminopyridine, 3,4-diaminopyridine, or clonazepam can be used to treat downbeat nystagmus. Memantine, 4-aminopyridine, and baclofen can be used to treat upbeat nystagmus. Gabapentin may help with torsional nystagmus [13]. Nystagmus can also be treated by injecting botulinum toxin into the muscles of the extraocular area or the retrobulbar area. The most frequent surgical methods are extraocular muscle recession, resection, tendon release, or lengthening, or a blend of these procedures may be used [14]. Electro-optical technologies are presently being developed to eradicate the visual symptoms of nystagmus without intrusive surgery [14].

Physical therapy treatment for nystagmus aids in gaze stabilization, ocular muscle strength improvement, and vestibular function maintenance [15]. This case report describes a classic example of nystagmus linked with albinism, as well as the patient's recovery with a well-planned physical therapy approach [16] and vestibular rehabilitation [17], as measured by the Vanderbilt Pediatric Dizziness Handicap Inventory for Patient Caregivers (DHI-PC) [18].

## Case presentation

Patient information

Due to aberrant continuous ocular movements and head tilting to one side while focusing on anything, a 10-year-old male child presented to the physiotherapy department with complaints of inability to concentrate on studying and writing. Since birth, he has been pre-diagnosed with OCA. The parents initially observed the motions when the infant was three months old. When the child was eight years old, his parents took him to a nearby hospital for an eye exam, and he was told he needed physiotherapy for nystagmus. His parents have then complained of symptoms of increased frequency and intensity of eye movements in their child. Ocular motions are enhanced during times of stress and reduced during times of relaxation. The child has myopia with a bilateral power of -2.50 D. He has no family history of nystagmus or albinism. Due to breech position and insufficient amniotic fluid, he was born through C-section and his mother's gravida, para, abortus, living (GPLA) score was G3P2L2A1. He met developmental milestones on schedule.

Clinical findings

The patient was conscious and oriented to time, place, and person, and his speech, language, intellect, and memory were all intact. According to the observation, it was the right beating nystagmus. There was no deformity, no muscular loss, and no edema in the leg. All superficial, deep, and cerebral sensations were normal on examination, and the limbs had normal tone bilaterally. His bilateral coordination was normal. The head impulse test (HIT) and head-shaking-induced nystagmus (HSN) both indicated that nystagmus was evident.

Timeline

The patient was diagnosed with a case of OCA in the year 2011. In the year 2012, his parents first noticed abnormal eye movements. An eye checkup was done on December 2, 2021, and was advised to do physiotherapy. Physiotherapy rehab was initiated the next day.

Diagnostic assessment

**Figure 1:**
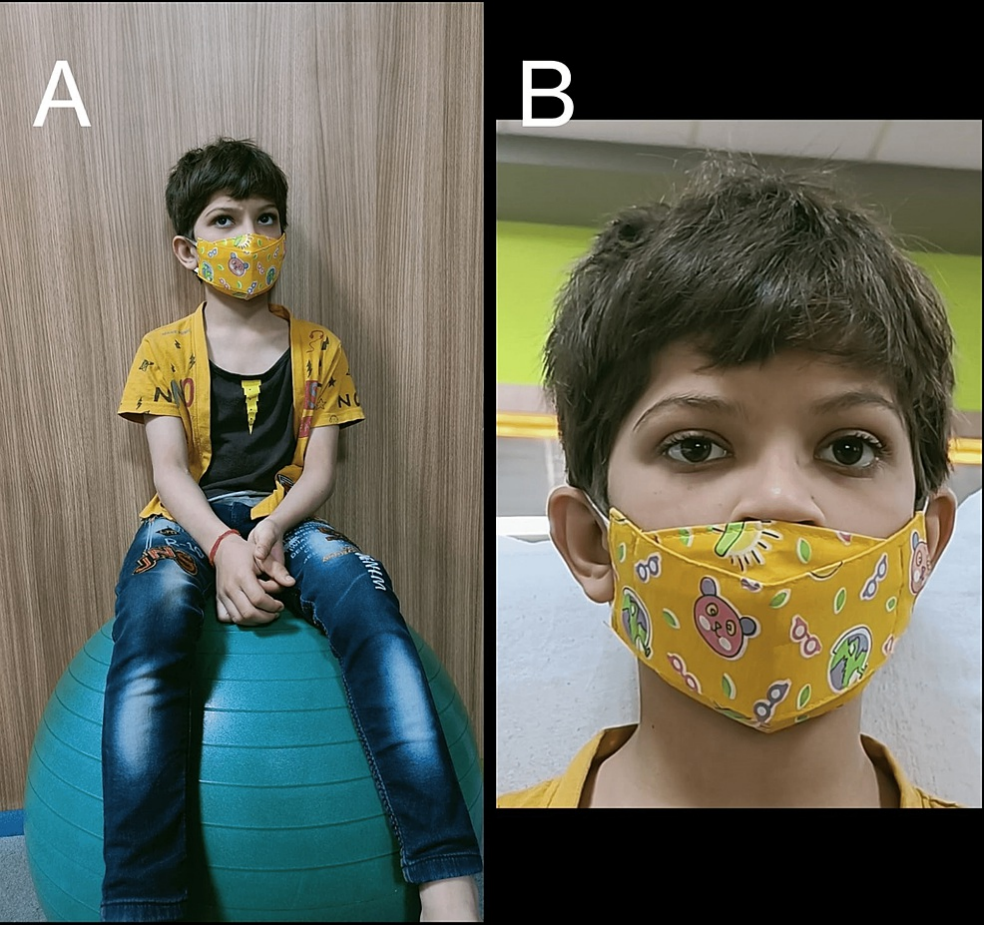
(A) Demonstrates the patient performing eye movements on a vestibular ball while sitting as a part of Cawthorne-Cooksey exercises. (B) Demonstrates the clinical image of the patient showing features of albinism.

On physical examination by the concerned pediatrician, he was diagnosed with OCA since birth (Figures 1A, 1B). The creamy-white and pale skin, light brown hair and eyes, and visual defect in the form of nystagmus made the clinical picture very clear. The parents later confirmed that the melanin pigmentation had increased gradually with time in the skin, hair, and eyes, but the nystagmus was still present.

Diagnosis

Nystagmus following oculocutaneous albinism.

Therapeutic intervention

Physiotherapy rehabilitation was continued for a month. Short-term goals are aimed at stabilizing gaze, reducing dizziness, reducing the frequency of eyeball movements, and increasing the strength of ocular muscles. Long-term goals* *are intended to maintain the strength of ocular muscles, maintain vestibular function, and improve the quality of life of the patient.

**Figure 2:**
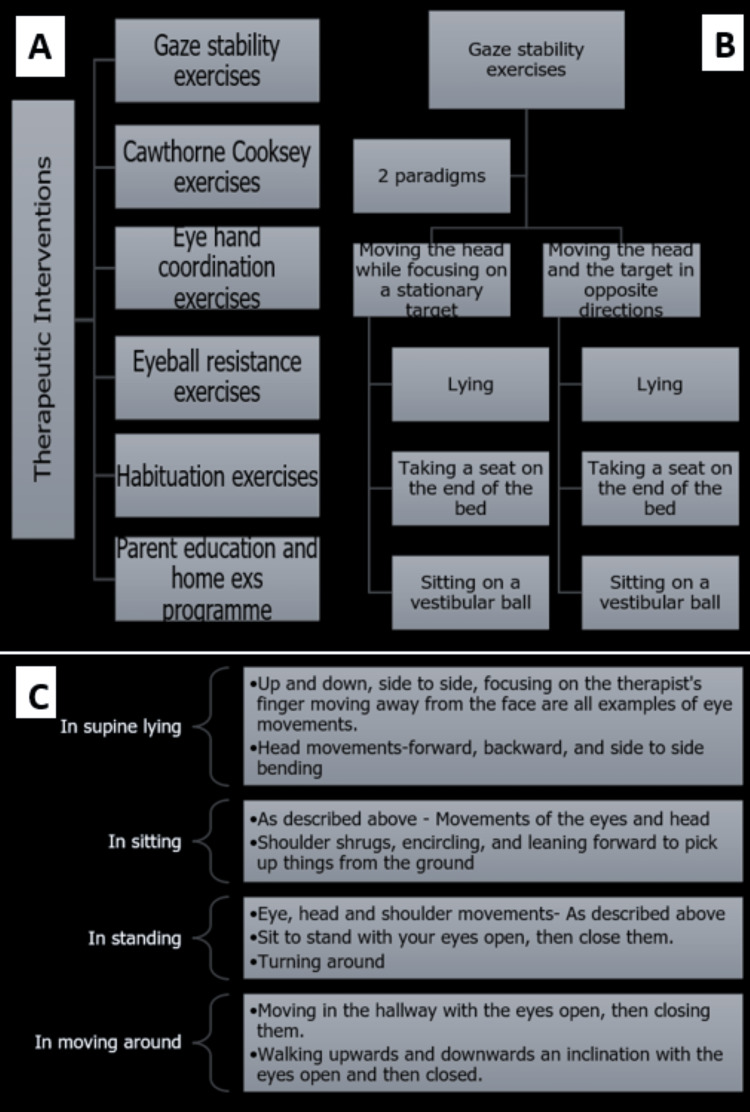
(A) Therapeutic exercises, (B) gaze stability exercises, and (C) Cawthorne-Cooksey exercises.

The physiotherapy interventions given to the patient included gaze stability exercises, Cawthorne-Cooksey exercises, eyeball resistance exercises, eye-hand coordination exercises, and habituation exercises (Figures 2A-2C). 

Gaze Stability Exercises

The purpose of these exercises is to strengthen the VOR and other systems that allow the head to maintain its gaze while moving. There are two ways you can use these exercises. In the first technique, the patient is told to move his head horizontally as quickly as he can while keeping his gaze fixed on a stationary object. The second strategy requires the patient and the goal to move in opposite directions. These exercises were first performed while lying down, then progressed to sitting at the edge of the bed, and then to sitting on a vestibular ball [15] (Figure 2B).

Cawthorne-Cooksey Exercises

In supine lying, these included eye movements upwards and downwards, from side to side and trying to focus on the therapist's forefinger moving from three to one foot away from the patient's face; and movements of the head bending forward-backward and then from side to side. Both movements start slowly, progressing to an increase in speed. In sitting, the movements of the eye and head as above, in addition to shoulder-shrugging and circling and bending forward to pick up objects from the level ground. In standing, eye, shoulder, and head movements as above, shifting from sitting to standing with eyes open, progressing to eyes closed, and turning around in between. In moving around in a room, it included walking across the room with eyes open progressing to eyes closed, and walking up and down a slope with eyes open progressing to eyes closed [19] (Figure 2C).

Eye-Hand Coordination Exercises

The goal of these exercises is to improve the coordination between the movements of the eye and hand. It included finger-to-nose, finger-to-finger, and finger-to-therapist’s finger exercises. Ten repetitions of each exercise have been given.

Eyeball Resistance Exercises

The goal of these exercises is to improve ocular muscle strength. The resistance is applied manually with the therapist’s finger. The patient is asked to move the eyeball in each of the eight directions, and the motion is resisted. Five repetitions in each direction are given bilaterally.

Habituation Exercises

It included tandem walking with visual feedback, starting with tandem walking with the abduction of both arms to 90 degrees and progressing to tandem walking with both arms by the side of the body.

Parent Education and Home Exercise Programs

It entailed informing the parents about the problem and the predicted prognosis. The exercises were taught to the parents as part of a home exercise regimen. The parent was taught to provide verbal cues to the child when necessary to correct the head tilting while focusing on something.

Follow-up and outcomes

**Figure 3:**
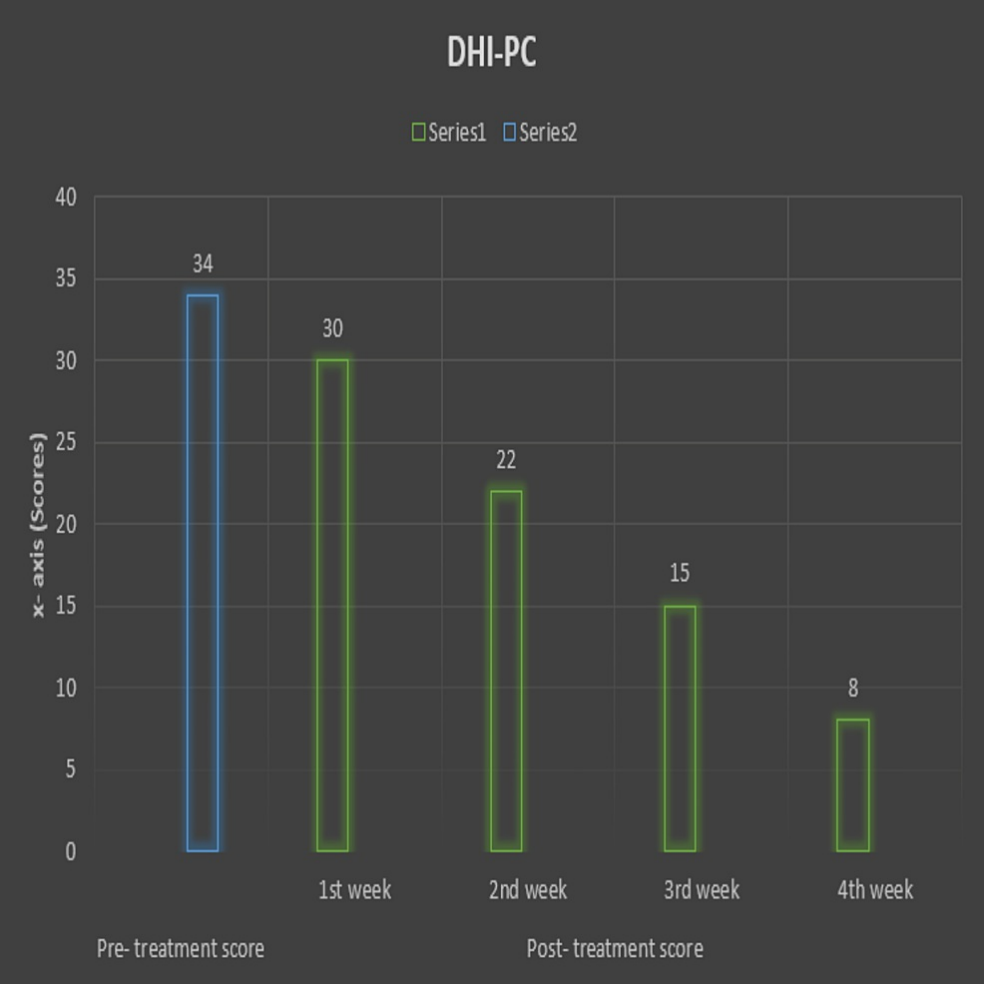
A bar diagram representing pre- and post-treatment scores on the DHI-PC. The X-axis indicates weeks of treatment. The Y-axis indicates the score. DHI-PC: Vanderbilt Pediatric Dizziness Handicap Inventory for Patient Caregivers.

Vanderbilt Pediatric Dizziness Handicap Inventory for Patient Caregivers (DHI-PC) [18]. The maximum score of the outcome is 84. The patient had significant relief from dizziness post-treatment. The pre- and post-treatment scores are represented on a bar diagram (Figure 3).

## Discussion

Albinism is a hereditary disorder that affects many organ systems. As a result, a multidisciplinary approach is the best option for dealing with the situation. In non-syndromic albinism patients, vision impairments such as nystagmus are the most common cause of infirmity. If not addressed early enough, delayed visual development might lead to academic difficulties. Social isolation and despair can be exacerbated by low self-esteem and social detachment. Attention deficit disorder is more common among people with albinism than in other races. To optimize results and minimize the social and educational consequences, they should be addressed as soon as possible [6].

Although there are now clinically validated pharmacological therapies for nystagmus, their use in children is still restricted. Furthermore, surgical methods to rectify a head turn in nystagmus are successful, as are surgeries to suppress nystagmus, such as the tenotomy of four muscles [8].

## Conclusions

This study concluded that after a month of rehabilitation, there was a significant decrease in ocular movements and head tilt while seeing and dizziness.

This case study throws light on the significance of a systematic physical therapy approach in dealing with cases of albinism-associated nystagmus and dizziness to ensure a good patient recovery.
